# Emergency esophagectomy: Experience of a high volume esophageal cancer center

**DOI:** 10.12669/pjms.39.2.6613

**Published:** 2023

**Authors:** Faizan Ullah, Awais Naeem, Nighat Bakhtiar, Osama Shakeel, Ihtisham-Ul-Haq, Shahid Khattak, Aamir Ali Syed

**Affiliations:** 1 Dr. Faizan Ullah, Fellow Surgical Oncology, Department of Surgical Oncology, Shaukat Khanum Memorial Cancer Hospital and Research Centre (SKMCH&RC), Lahore, Pakistan; 2 Dr. Awais Naeem, Fellow Surgical Oncology, Department of Surgical Oncology, Shaukat Khanum Memorial Cancer Hospital and Research Centre (SKMCH&RC), Lahore, Pakistan; 3 Dr. Nighat Bakhtiar, Fellow Surgical Oncology, Department of Surgical Oncology, Shaukat Khanum Memorial Cancer Hospital and Research Centre (SKMCH&RC), Lahore, Pakistan; 4 Dr. Osama Shakeel, Resident General Surgery, Department of Surgical Oncology, Shaukat Khanum Memorial Cancer Hospital and Research Centre (SKMCH&RC), Lahore, Pakistan; 5 Dr. Ihtisham-Ul-Haq, Fellow Surgical Oncology, Department of Surgical Oncology, Shaukat Khanum Memorial Cancer Hospital and Research Centre (SKMCH&RC), Lahore, Pakistan; 6 Dr. Shahid Khattak, Consultant Surgical Oncologist, Department of Surgical Oncology, Shaukat Khanum Memorial Cancer Hospital and Research Centre (SKMCH&RC), Lahore, Pakistan; 7 Dr. Aamir Ali Syed, Consultant Surgical Oncologist, Department of Surgical Oncology, Shaukat Khanum Memorial Cancer Hospital and Research Centre (SKMCH&RC), Lahore, Pakistan

**Keywords:** Emergency esophagectomy, Perforation, Mortality

## Abstract

**Objective:**

The objective of the study was to review the experience of dealing oncological emergency esophagectomies at a dedicated Cancer hospital.

**Methods:**

We performed a retrospective review of data of eleven esophagectomies at the Department of Surgical Oncology, Shaukat Khanum Memorial Cancer Hospital and Research Centre, Lahore (SKMCH&RC) Pakistan, from 1^st^ January, 2009 to 30^th^ June, 2019. Out of 590 oncological esophagectomies, eleven patients had emergency resection. We collected the data of demographics, primary disease, comorbidities, location of tumor and perforation, cause of perforations, radiological and endoscopic findings, clinical findings and follow-up visits after discharge. Data was analyzed by SPSS version 21 for windows.

**Result:**

All 11 patients out of five hunded ninety had esophageal cancer. At the time of initial staging, eight (72%) had locally advanced stage (stage III and IV). Open transhiatal approach was used in six (55%) patients, and the rest had three stage esophagectomies. Primary reconstructions with gastric conduit were performed in all, except in two (18%) patients, Respiratory complications were the most common of the encountered complications, seven (63%) of the patients had palliative resection. Ninety day mortality was observed in 3(27.3%) patients. On long term follow up, six patients had recurrence, with median Disease-Free Survival (DFS) 5.88 months and Median Overall Survival (OS) was 6.37 months. Out of 11, only three patients are alive without disease, while one patient is lost during follow-up.

**Conclusion:**

Emergency esophagectomy is a lifesaving procedure; there should be multidisciplinary team approach towards the management. Early diagnosis and management is of paramount importance.

## INTRODUCTION

Despite increasing Incidence of esophageal perforation due to generalization and popularity of endoscopy and ongoing advancements in endoscopic interventions,^1^-^3^ literature regarding surgical experience of emergency esophagectomies remains truncated.

Emergency esophagectomy is usually performed for esophageal perforation secondary to iatrogenic perforation, spontaneous perforation (Boerhaave syndrome) or rarely tumor related causes like spontaneous perforation and bleeding.^4^ Regardless of the cause, esophageal perforation has significantly high mortality; ranging from 10-25% even if managed promptly within 24 hours^5^,^6^ or it could be as high as 67% if managed late. Due to atypical presentation, up to 50% of patients receive treatment after 24 hours.^7^ Diagnostic error in detection and lack of clear treatment guidelines attributes to further delayed diagnosis and high mortality.

Improved outcomes have been attributed to early detection, iatrogenic perforation during endoscopy versus spontaneous perforation, no underlying disease, and most importantly availability of multidisciplinary team, experienced in esophageal surgeries.^8^-^11^ There is an on-going debate about the definitive treatment which ranges from conservative treatment to emergency esophagectomy with or without reconstruction. Some authors do emphasize the eligibility criteria for resections, which includes presentation after more than 24 hours, underlying obstructive disease, esophagus not salvageable due to extensive perforation, widespread mediastinitis and pleuritis.^12^-^15^

Shaukat Khanum Memorial Cancer Hospital and Research Centre (SKMCH&RC) is a specialized cancer hospital that receives high volume of esophagogastric cancers. Nearly 600 esophagectomies have been performed for esophageal cancers in the last ten years. We present our experience of eleven emergency esophagectomies including their outcomes. Attention has been paid to the important publications on the management of esophageal perforation by surgeons who have command on this subject.

## METHODS

This is a retrospective study with convenient sampling done at the Department of Surgical Oncology, (SKMCH&RC) Lahore, Pakistan. After obtaining institutional approval for the study (Ref No.: EX-22-02-20-01-A1; dated: February 25, 2022), patients from 1^st^ January 2009 to 30^th^ June, 2019 were included. Number of esophagectomies performed during the afore mentioned period were 590. Out of which 11 patients had emergency esophagectomies for different reasons. Data based on demographics, primary disease, comorbidities, location of perforation, reasons of perforation, radiological and endoscopic findings, presence of pleuritis or mediastinitis and hemodynamic stability at the time of presentation and follow-up visits was recorded from the Hospital Information System (HIS). Other variables which were exclusively looked for were Charlson Comorbidities Index Score, any recent chemotherapy (within four weeks), time from perforation to surgical management (>24 hours).

Data was entered and analyzed into SPSS version 21. Descriptive statistics were calculated. Mean±SD, median or frequencies were calculated for clinicopathological variables, management approaches, inpatient outcomes and mortality. Various variables were analyzed to observe their impact on mortality and overall survival. For this Fisher exact test or t-test was applied to compare the groups at 95% confidence interval and P-value of ≤0.05 was taken as statistically significant.

## RESULTS

Out of all 590 esophagectomies performed during the ten-year study period, only 11 were emergency esophagectomies (Table-I). Most common cause of surgery was iatrogenic perforation (8 patient), two patients had spontaneous perforation of esophagous while only one patient had retractable bleeding tumor.

**Table-I:** Complete Population Details.

	Gender	Age	Diagnosis	Duration to Surgery	Vasopressors	Reason of Surgery	Site of perforation (esophagus)	Type of Surgery	Type of Resection	Anastomosis	Clavien Dindo Grading	TNM	90 Day Mortality
1	Female	48	Well differentiated Squamous cell carcinoma	<24 h	No	Perforation during endoscopy	Middle	Open three stage esophagectomy	R2	Yes	1	P4bN1	No
2	Female	45	Moderately differentiated Squamous cell carcinoma	<24 h	Yes	Perforation during endoscopy	Lower	Open Transhiatal esopahagectomy	R1	Yes	3b	Pt3N2	No
3	Male	50	Poorly differentiated Squamous cell carcinoma	>24 h	No	Spontaneous Rupture of esophagus	Middle	Open Transhiatal esopahagectomy	R0	Yes	5	Pt1bN0	Yes
4	Female	65	Moderately differentiated Squamous cell carcinoma	<24 h	No	Perforation during endoscopy	Lower	Open Transhiatal esopahagectomy	R0	Yes	3a	Pt3N1	No
5	Female	65	Moderately differentiated Squamous cell carcinoma	<24 h	Yes	Perforation during endoscopy	Middle	Minimally invasive three stage esophagectomy	R2	Yes	2	Pt3N0	No
6	Female	61	Moderately differentiated Squamous cell carcinoma	<24 h	No	Perforation during endoscopy	Lower	Minimally invasive three stage esophagectomy	R0	Yes	1	Pt2N3	No
7	Male	40	Moderately differentiated Squamous cell carcinoma	<24 h	No	Perforation during endoscopy	Lower	Minimally invasive three stage esophagectomy	R2	Yes	1	Pt3N1	No
8	Male	47	Moderately differentiated Squamous cell carcinoma	>24 h	Yes	Perforation during endoscopy	Lower	Open Transhiatal esopahagectomy, esophagostomy and gastrostomy	R0	No	5	Pt3N0	Yes
9	Male	24	Moderately differentiated Squamous cell carcinoma	<24 h	No	Perforation during endoscopy	Middle	Open Transhiatal esophagectomy	R2	Yes	1	Pt2N0	No
10	Female	37	Moderately differentiated Squamous cell carcinoma	>24 h	Yes	Spontaneous Rupture of esophagus	Lower	Minimally invasive three stage esophagectomy	R1	No	5	Pt3N0	Yes
11	Male	39	Moderately differentiated Squamous cell carcinoma	<24 h	No	Bleeding tumor	Lower	Lap converted to open Transhiatal esophagectomy	R2	Yes	4a	Pt4aN1	No

### 1. Clinicopathological characteristics of patients:

Although we routinely encounter all histological types, but in our study squamous cell carcinoma were noticed in all the 11 patients with esophageal cancer. At diagnosis, most patients (n=8) had locally advanced stage at the time of initial staging (stage III and IV). Distant metastasis was not noted in any patient. The other characteristics are given in Table-II.

**Table-II:** Clinicopathological features

Characteristics	Results	Statistics
Age (years)	47.36 (± 12.64)	Mean(±SD)
Male: Female	0.8 : 1	Ratio
BMI	19.65 ± 4.63	Mean(±SD)
***ECOG***		
0	5	Frequency
1	6	
***Reason for surgery***		Frequency
Perforation during endoscopy	8	
Spontaneous perforation	2	
Bleeding tumor	1
Time from perforation to surgery (hours)	8 (3-5)	Median
***T Staging***		Frequency
T2	1	
T3	7	
T4a	2	
T4b	1	
***N staging***		Frequency
N0	3	
N1	7	
N2	1	
***Histological differentiation***		Frequency
Well	1	
Moderate	9	
Poor	1	
Patients received Neoadjuvant chemotherapy	4	Frequency

### 2. Management approaches and In-hospital outcomes:

In Term of surgical management, open trans hiatal approach was used in six patients, and the rest had three stage esophagectomy. Primary reconstructions with gastric conduit were performed in all patients except in two patients in whom the reconstruction was deferred due to general condition and conduit necrosis. For reconstruction all patients in whom anastomosis was done single layered, interrupted, hand sewn, end to side anastomosis was performed in the neck.

Eight (72%) patients received peri-operative blood transfusion and 4(36%) patients required intra-operative vasopressor support. Mean Operative Time (SD) was 245±111 minutes. Median Hospital Stay was 9 (6-55) days and Median ICU stay was 2(1-55) days. Respiratory complications were the most common complication encountered, requiring intubation more than two weeks in 2(18%) of the patients, additional post-operative chest tube insertion either for pleural effusion or pneumothorax in 3(27%) and bronchoscopy for mucous plug removal in 2(18%) patients. Another 2 (18%) patients required re-intervention in the form of thoracotomy for primary hemorrhage and wound debridement and esophagostomy refashioning respectively. Two or more ClavienDindo Grades were noted in seven patients. Unfortunately, 7(63%) patients had palliative resection 5(41%) had R2 resection, 2(18%) R1 resection)

### Survival outcomes:

None of the patients had 30-day mortality. However, mortality during 90 days was observed in 3(27.3%) patients. Various categorical variables which were analyzed to observe their impact on 90 days mortality are shown in Table-III, but no statistical significance was found in all cases except the duration between perforation and surgery (p-value 0.006). Furthermore, no statistical significance was found when t-test was applied to see impact of total number of hospital days and total ICU days on 90 days mortality (p-values 0.340 and 0.320 respectively).

**Table-III:** Impact on 90 days mortality

Factors		90 days mortality		P-value

		Yes	No	
Gender	Female	1	5	0.545
	Male	2	3	
	Total	3	8	
Age Groups	<47 Years	1	4	1.000
	>47 Years	2	4	
	Total	3	8	
Neoadjuvant chemotherapy	Yes	2	2	0.491
	No	1	6	
	Total	3	8	
Tumor differentiation	Well	0	1	0.491
	Moderate	2	7	
	Poor	1	0	
	Total	3	8	
Co- morbid	No	0	1	0.618
	IHD	0	3	
	Hypertension	3	4	
	Total	3	8	
Time to surgery	<24 hours	0	8	0.006
	>24 hours	3	0	
	Total	3	8	
Vasopressors	Yes	2	1	0.07
	No	1	7	
	Total	3	8	

On long term follow up, six patients had recurrence, with median Disease-Free Survival (DFS) 5.88 months, out of which five patients had both loco-regional and distant metastasis, while only one patient had local recurrence. Median Overall Survival (OS) was 6.37 months. Out of 11, only three patients are alive without disease, while one patient has been lost from follow up. No statistical significance was observed when we analyzed impact of age (p-value 0.556 and correlation -200) and gender (p-value 0.738) on overall survival.

## DISCUSSION

Iatrogenic esophageal perforation during endoscopy was the most common indication of emergency esophagectomy at our center in the studied cohort of patients. Spontaneous Perforation secondary to esophageal cancer is very rare and accounts for only 1% of all esophageal perforations.^16^ We encountered two cases of spontaneous tumor perforation. Tumor bleeding is a very rare phenomenon in esophagus cancers, reported cases only accounts for massive bleeding due to aorto-esophageal fistula secondary to tumor invasion.^17^ One of our patients had locally advanced bleeding tumor and underwent emergency esophagectomy as endoscopic control of bleeding failed to achieve.

As our institute is a dedicated cancer hospital, we only deal with patients with esophageal cancer, and benign esophageal pathologies requiring surgery are not managed at our hospital. All of the patients in our study had locally advanced tumors therefore, we encountered significantly high mortality and morbidity. Michel and Garilo reported mortality of 23% in patients with underlying malignancy.^18^ Mortality rate in our patients was 27.3% (n=3). Delay in treatment is an established risk factor for increased mortality and morbidity.^19^ In our experience, all patients (n=3) who presented after 24 hours of perforation died within 90 days of intervention.

Decision of surgical intervention for esophageal perforation is difficult and requires precise surgical judgment based on following points; iatrogenic versus spontaneous perforation, location of perforation, underlying esophageal pathology, time from perforation to presentation, general health of the patient at the time of perforation and lastly presence of sepsis, mediastinitis, pleuritis secondary to contamination caused by perforation.^20^

Several surgical options from primary repair, reinforcement technique(with pericardial, intercostal muscle flaps, diaphragmatic or gastric flaps), controlled fistula formation and definitive esophagectomy with or without reconstruction have been used effectively.^21^,^22^ Although endoluminal therapy like stenting challenges this perception but surgery with esophagectomy remains pivotal for definitive treatment.^23^-^25^ Regardless of the intervention, optimal and prompt emergency room optimization and resuscitation is vital in final outcome of patient.

Surgical intervention with resection of distal obstruction is vital for better short-term outcome.^26^-^28^ Esophagectomy with reconstruction using gastric tube was performed in 9 (81%) patients while esophagectomy with cervical esophagostomy was performed in 2(18%) patients. Karen suggested similar approach if perforation is not suitable for repair or reconstruction after resection, however he recommended that stoma should be formed in left anterior chest wall rather than neck for better control.^29^ Yeo at el. suggested esophageal resection via transhiatal approach for perforated esophagus.^30^ However, it is inevitable to avoid transthoracic approach for tumor and perforation located in mid thoracic esophagus. Remedy to this challenge, we found that in experienced hands minimal invasive esophagectomy via VATS and laparoscopy are equally effective. Emergency esophagectomy is a lifesaving palliative procedure, as only four out of eleven patients achieved R0 resection. Median DFS and OS are significantly lower compared to elective procedure (DFS: 5.88vs10.08; OS: 6.37vs18.04 months respectively).

### Limitations

The limitation of this study is its retrospective nature. The sample size isn’t large enough to make recommendations about the management of esophageal perforation. However, it does give us information about the poor prognosis of esophageal tumor perforations irrespective of the type of intervention and the post-operative morbidity after surgical intervention. This is first such experience being published from Pakistan and will, hopefully, pave way for future research on this topic.

## CONCLUSION

Esophageal perforation is a challenging pathology with no management standardization. Decision of Emergency esophagectomy after esophageal tumor perforation is debatable and should be individualized approach to ensure the best results; to obtain best outcome there should be multidisciplinary team approach with early diagnosis and management is of paramount importance.

### Authors’ Contributions:

**FU:** Study lead, study concept and manuscript writing.

**AN:** Study lead and manuscript writing.

**NB:** Conception and design.

**OS:** Data collection and drafting the manuscript.

**IH:** Data Collection and analysis of data.

**SK:** Critical Revision for important intellectual content.

**AAS:** Final approval of the Manuscript.
